# Endostatin/Collagen XVIII Is Increased in Cerebrospinal Fluid after Severe Traumatic Brain Injury

**DOI:** 10.1155/2013/402375

**Published:** 2013-09-08

**Authors:** Hao Chen, Li-Xia Xue, He-Li Cao, Shi-Wen Chen, Yan Guo, Wen-Wei Gao, Shi-Ming Ju, Heng-Li Tian

**Affiliations:** ^1^Department of Neurosurgery, Shanghai Sixth People's Hospital, Shanghai Jiaotong University, 600 Yishan Road, Shanghai 200233, China; ^2^Department of Neurology, Shanghai Sixth People's Hospital, Shanghai Jiaotong University, Shanghai 200233, China

## Abstract

Recent studies have suggested that endogenous angiogenesis inhibitor endostatin/collagen XVIII might play an important role in the secondary brain injury following traumatic brain injury (TBI). In this study, we measured endostatin/collagen XVIII concentrations serially for 1 week after hospitalization by using the enzyme-linked immunosorbent assay method in the cerebrospinal fluid (CSF) of 30 patients with TBI and a Glasgow Coma Scale (GCS) score of 8 or less on admission. There was a significant trend toward increased CSF levels of endostatin after TBI versus control from 72 h after injury. In patients with GCS score of 3–5, CSF endostatin concentration was substantially higher at 72 h after injury than that in patients with GCS score of 6–8 (*P* < 0.05) and peaked rapidly at day 5 after injury, but decreased thereafter. The CSF endostatin concentration in 12 patients with an unfavorable outcome was significantly higher than that in 18 patients with a favorable outcome at day 5 (*P* = 0.043) and day 7 (*P* = 0.005) after trauma. Receiver operating characteristic curve analysis suggested a reliable operating point for the 7-day CSF endostatin concentration predicting poor prognosis to be 67.29 pg/mL. Our preliminary findings provide new evidence that endostatin/collagen XVIII concentration in the CSF increases substantially in patients with sTBI. Its dynamic change may have some clinical significance on the judgment of brain injury severity and the assessment of prognosis. This trial is registered with the ClinicalTrials.gov Identifier: NCT01846546.

## 1. Introduction

 Angiogenesis following traumatic brain injury (TBI) is not only critical to the posttraumatic tissue reparative processes and restoration of function, but is also associated with the development of secondary brain injury [1–3]. It is characterized by vessel sprouting and arborisation reaching maximum levels 3–5 days after TBI [4, 5] and is regulated by pro- and antiangiogenesis factors [6]. Currently, expressions of several proangiogenic factors and their neuroprotective effects following TBI have been reported in different experimental animal models and in vivo human studies [7–9]. However, there is little data on what effects antiangiogenic factors will have on healing wounds, especially in TBI patients. Endostatin/collagen XVIII is one of the most potent endogenous angiogenesis inhibitors and was reported to be expressed by endothelium and activated microglia/macrophages and plays a role in the development of the second brain damage after experimental TBI [10, 11]. Although the increase of endostatin/collagen XVIII^+^ macrophages/microglial cells has been reported in patients with traumatic brain injury [12], nothing is known about changes in cerebrospinal fluid (CSF) endostatin/collagen XVIII concentrations. We hypothesized that the role of endostatin/collagen XVIII as an important factor in the response to acute brain trauma would be reflected by alterations in CSF endostatin/collagen XVIII concentration and that these alterations may correlate with the severity of the injury and with the outcome. Therefore, we examined the concentrations of endostatin/collagen XVIII in the CSF of noninjured controls and the changes in patients with severe TBI during early posttraumatic period, to determine whether differences exist, and assessed the relation of CSF endostatin/collagen XVIII to the injury severity and prognosis of such patients with TBI.

## 2. Patients and Methods

### 2.1. Patient Population

Between October 2006 and March 2007, we conducted a prospective cohort observational study of patients with severe TBI (Glasgow Coma Scale (GCS) score of 8 or less) requiring continuous lumbar drainage of CSF in the neurosurgery ward of the Sixth People's Hospital affiliated to Shanghai Jiaotong University. Patients delivered within 4 h whose highest abbreviated injury score (AIS) was 3 or less (other than head injury) were considered to be isolated TBI cases and were included. To avoid interfering factors, patients who suffered open or combined injuries or had existing prior neurological disease were excluded. Those who died within the first week of hospitalization and/or whose serial CSF samples could not be obtained were also excluded as having incomplete data. Accordingly, 30 patients were analyzed in this study. Demographic data, including age, gender, mechanism of trauma, and GCS score at admission, were documented when the patients arrived at the emergency room. The control group comprised 20 patients whose CSF was examined via lumbar puncture for investigation of suspected neurological disease. All patients had normal neurological examination and negative imaging studies, and none had evidence of trauma or preexisting neurological disease, including tumors, vascular anomalies, or abnormalities of CSF. The study had approval from the hospital ethics committee and an informed consent for participating in the study was obtained from an appropriate member of each patient's family before performance of lumbar drainage.

### 2.2. Patient Management

Immediately after admission, all patients were evaluated with an initial CT scan and were followed with serial neurological examinations. All the imaging studies were technically adequate and were reviewed by the staff of the radiology department. Subsequently, insertion of a lumbar drain into the subarachnoid space was conducted in a standard fully sterile technique. The lumbar subarachnoid catheter was placed in the subarachnoid place with 15 cm in length via lumbar 4-5 or 3-4 interspinous space through a spinal needle, using routine puncture procedure with the patient in lateral decubitus position. The remaining part of the catheter was positioned to course transversely across the back and slightly up the side of the abdomen. The catheter insertion site was treated with povidone-iodine ointment and the entire catheter was covered with drape. The distal end of the catheter was hooked up to an empty transfer pack with a macrodrip chamber that allows accurate quantification of CSF drained. This system used a standard lumbar drain connected to an intravenous infusion pump to provide drainage of CSF in a constant and predictable manner. The patients were kept at absolute bed rest but allowed to turn from side to side and could not sit up to about 45° angle in bed. The drainage chamber was adjusted with respect to the patients head so that the drainage rate could be altered and overdrainage could be prevented. The desired drainage rate was approximately 5–15 mL/hour or 120–360 mL/day. The system was left in place for 7–10 days. At the end of the course of external drainage, the drain was left closed. The development of any neurological findings should prompt rapid discontinuation of lumbar drainage and immediate radiographic evaluation. There was no evidence of complications, such as tension pneumocephalus, brain herniation, and intracranial infection. All patients were evaluated and treated according to the guidelines of the “Management of Severe Head Injury” in the neurosurgery intensive care unit [13]. Outcome was evaluated using the Glasgow Outcome Scale (GOS) 6 months after the trauma: GOS 1 = death; GOS 2 = vegetative state; GOS 3 = severe neurological deficit; GOS 4 = mild neurological deficit; and GOS 5 = premorbid level of functioning or complete recovery [14]. For statistical comparison, unfavorable outcome was defined as a GOS score of ≤3, and favorable outcome was defined as a GOS score of >3. Surviving patients participated in follow-up interviews either by telephone or in person at the clinic. 

### 2.3. Determination of CSF Endostatin/Collagen XVIII Concentrations

CSF samples from patients with severe TBI were collected serially from the lumbar drainage system at 1, 3, 5, and 7 d after injury. CSF samples from the controls were also collected from the lumbar subarachnoid space. Samples were centrifuged to remove cellular debris, and the supernatant was immediately frozen at −20°C after sampling and stored for later analysis. 

Endostatin/collagen XVIII concentrations in CSF were determined by enzyme-linked immunosorbent assay (ELISA) with commercially available kits (RapidBio. Lab, California, USA), according to the manufacturer's instructions. This assay employs the quantitative sandwich enzyme immunoassay technique. In brief, a monoclonal antibody specific for endostatin/collagen XVIII had been pre-coated onto a microplate. Standards and CSF samples were pipetted into the wells and any endostatin/collagen XVIII present was bound by the immobilized antibody. An enzyme-linked monoclonal antibody specific for endostatin/collagen XVIII was added to the wells. Following a wash to remove any unbound antibody-enzyme reagent, a substhumane solution was added to the wells and color developed in proportion to the amount of endostatin/collagen XVIII bound in the initial step. After addition of stop solution into each well, the color development was stopped and the optical density was measured within 30 minutes, using a microplate reader set to 450 nm. No significant cross-reactivity or interference was observed in this assay. Finally, a standard curve was constructed by plotting the mean absorbance for each standard on the *y*-axis against the concentration on the *x*-axis and a best fit curve was drawn through the points on the graph. The data may be linearized by plotting the log of the endostatin/collagen XVIII concentrations versus the log of the optical density, and the best fit line can be determined by regression analysis. Consequently the endostatin/collagen XVIII concentrations in CSF samples were obtained by reading form the standard curve.

### 2.4. Statistical Analysis

The statistical package Statistical Program for Social Sciences (version 17.0; SPSS, Inc., Chicago, IL 60606-6412, USA) was used for analyses. All values are expressed as mean ± standard error of the mean (SEM) unless otherwise specified. A two-tailed Student's *t*-test was used for comparisons between TBI and control patients. The CSF concentrations were not normally distributed; therefore, group means were compared using the Mann-Whitney test. Fisher's exact test was used to compare proportions where appropriate. Statistical significance was assumed for *P* values of less than 0.05. A receiver operating characteristic (ROC) curve was used to assess the accuracy of predictions of poor outcome from the CSF endostatin/collagen XVIII concentrations. Using the ROC curve, a CSF endostatin/collagen XVIII concentrations cutoff value and its confidence in prognosis could be estimated based on the area under the ROC curve (AUC).

## 3. Results

### 3.1. Patients' Characteristics

Of the 30 patients with severe TBI, twenty were male and ten were female. The patients' age ranged from 16 to 82 years, with mean age of 51.1 years. The mechanisms of trauma included motor vehicle collisions, falls, heavy strikes (patients who were hit by heavy objects such as bricks, sticks, or falling objects), and assaults. Types of lesions, as evidenced by radiologic and neurologic symptoms or signs, included cerebral contusions/lacerations, intracranial hematomas, brain stem injury, and diffuse axonal injury. The control group contained thirteen male and seven female. Their age averaged 52.1 years and ranged from 34 to 66 years. Clinical characteristics of the two groups are summarized in Table 1. There were no significant differences between the severe TBI group and the control group in age or sex ratio.

### 3.2. Serial Changes of CSF Endostatin/Collagen XVIII Concentrations in Patients with Severe TBI

**Figure 1:**
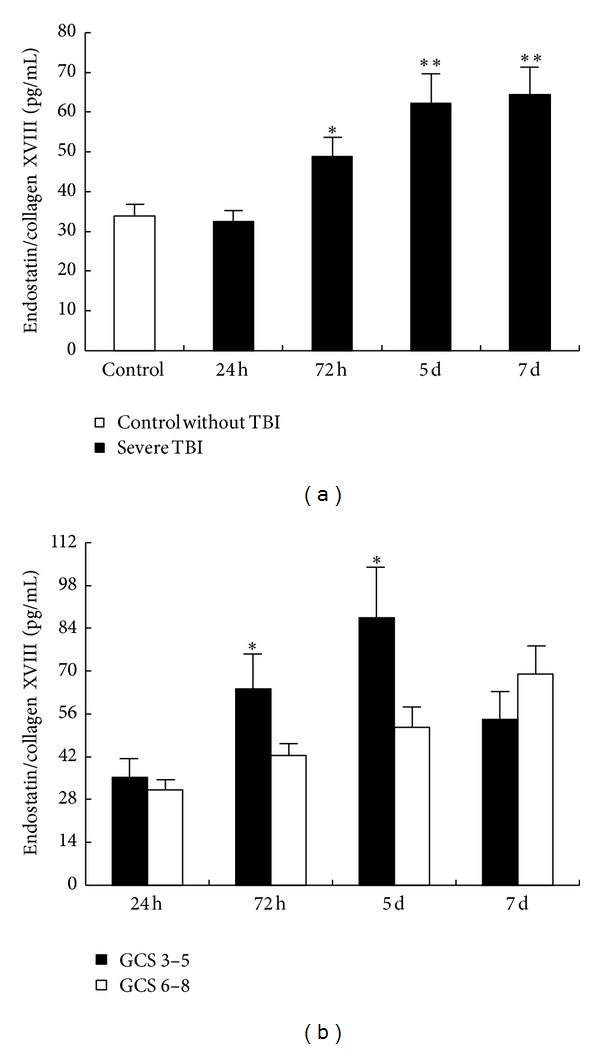
Elevation of CSF endostatin/collagen XVIII concentrations following TBI at days 3, 5, and 7 but not day 1. (a) Bar graph showing the time course of endostatin/collagen XVIII concentrations in CSF of severe TBI patients 1 day, 3 days, 5 days, and 7 days after injury, compared with that in control patients without TBI. (b) Bar graph showing CSF endostatin/collagen XVIII concentrations in two GCS score groups on days 1, 3, 5, and 7 after injury. Each bar represents the mean and standard error of the mean of all samples over all time points. Statistical analysis was performed by Student's *t*-test or Mann-Whitney test. *P < 0.05 and **P < 0.01 compared with their respective controls.

The mean CSF endostatin/collagen XVIII concentration of the control patients was 33.86 ± 2.97 pg/L. After TBI, there was a trend toward increased endostatin/collagen XVIII concentrations in CSF during our observed time period (Figure 1(a)). Although an obvious change of CSF endostatin/collagen XVIII concentration was not observed until 24 h after injury (32.48 ± 2.82 pg/mL, *P* > 0.05), its levels in CSF rapidly increased thereafter (Figure 1(a)). Elevation of endostatin/collagen XVIII concentration became significant at day 3 (48.96 ± 4.71 pg/mL, *P* < 0.05) and reached almost twice that of controls at day 5 (62.32 ± 7.32 pg/mL, *P* < 0.01) and day 7 (64.52 ± 7.02 pg/mL, *P* < 0.01) (Figure 1(a)).

### 3.3. Correlation of GCS Score with CSF Endostatin/Collagen XVIII Concentrations

Based upon the best recorded GCS within 24 h of admission, the patients were divided into severe TBI (GCS 6–8) (14 males, 7 females, and mean age of 49.7 years) and extra severe TBI (GCS 3–5) (6 males, 3 females, and mean age of 54.4 years) groups. These two groups did not differ significantly in age or sex ratio, nor was a significant difference observed in the mean CSF endostatin/collagen XVIII concentration at 24 h after injury between the two groups (35.27 ± 6.15 pg/mL versus 31.28 ± 3.12 pg/mL, *P* > 0.05) (Figure 1(b)). In patients with GCS 3–5, CSF endostatin/collagen XVIII concentration was substantially higher at 72 h after injury than that in patients with GCS 6–8 (64.17 ± 11.6 pg/mL versus 42.45 ± 3.95 pg/mL, *P* < 0.05), and it peaked rapidly at a mean value of 87.47 ± 16.32 pg/mL at day 5 after injury, approximately 1.5 times that in patients with GCS 6–8 (51.54 ± 6.77 pg/mL, *P* < 0.05) but declined to 54.09 ± 9.09 pg/mL 7 days after trauma, not significantly lower compared with the GCS 6–8 group at day 7 (68.99 ± 9.20 pg/mL, *P* > 0.05) (Figure 1(b)).

### 3.4. CSF Endostatin/Collagen XVIII Concentrations and Outcome

**Figure 2:**
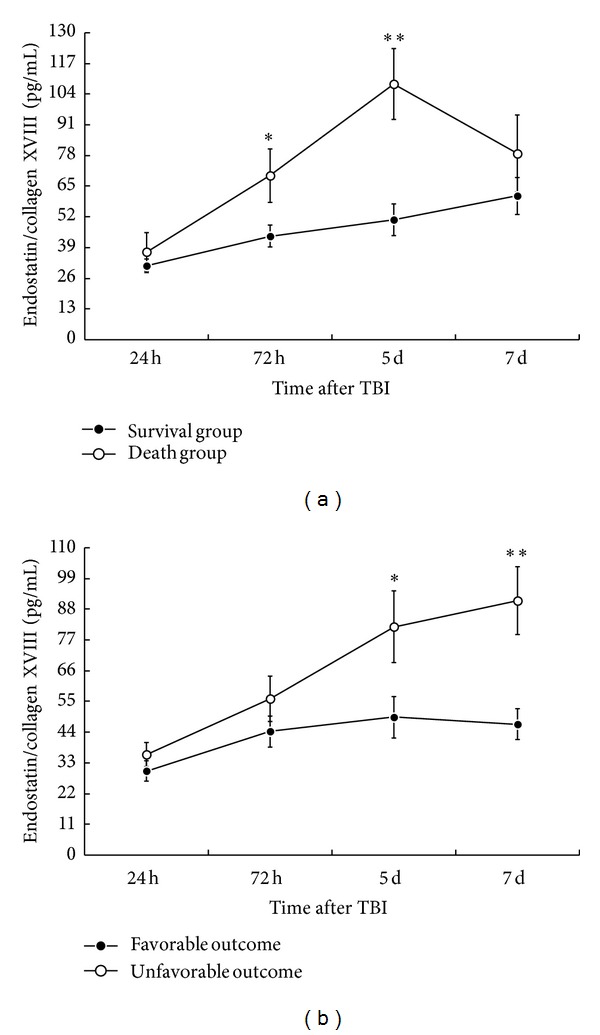
Graphs showing correlations between endostatin/collagen XVIII concentrations in CSF and 1-month prognosis situation and 6-month outcome in the 30 study patients with severe TBI. Data are expressed as mean ± SEM values. *P < 0.05 and **P < 0.01 compared with their respective controls.

Based on prognostic situation after a one month treatment, these patients were divided into two groups: survival group and death group (survival group: 15 males, 9 females, average age of 49.5 years; death group: 5 males, 1 female, average age of 57.3 years). Death group showed a trend toward increased CSF concentrations of endostatin/collagen XVIII after TBI versus survival group, which was especially significant at day 3 (*P* = 0.026) and day 5 (*P* = 0.001), although this did not reach statistical significance at 24 h and 7 days after trauma (Table 2 and Figure 2(a)).

**Figure 3:**
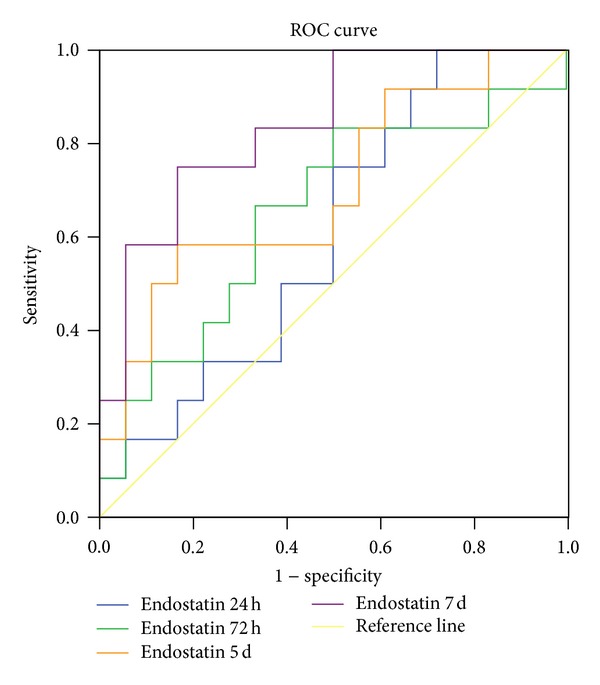
Receiver operating characteristic (ROC) curve to predict the possibility of unfavorable outcome at 6 months after trauma by measuring the CSF levels of endostatin/collagen XVIII. The value of 67.29 pg/mL at day 7 was considered the cutoff point, with the sensitivity and specificity of 75.0% and 83.3%, respectively.

At 6 months after TBI, outcome was favorable for 18 patients (11 males, 7 females, average age of 46.3 years) but unfavorable for twelve patients (9 males, 3 females, average age of 58.3 years). No significant difference was found between these two groups in age or sex ratio. The CSF endostatin/collagen XVIII concentration was significantly higher in patients with an unfavorable outcome than in patients with a favorable outcome at day 5 (*P* = 0.043) and day 7 after trauma (*P* = 0.005) (Table 3 and Figure 2(b)). No differences were found in CSF concentrations of endostatin/collagen XVIII between the two outcome groups at 24 h (*P* = 0.316) and 72 h (*P* = 0.230) after injury (Table 3 and Figure 2(b)). Using the ROC and AUC, we found a reliable operating point for the 7-day CSF endostatin/collagen XVIII concentration to be 67.29 pg/mL, with a sensitivity of 75.0% and a specificity of 83.3% (Figure 3); the AUC was 0.843 (*P* = 0.002, 95% confidence interval 0.701–0.984, Table 4). There were 12 patients who had a CSF endostatin/collagen XVIII level higher than 67.29 pg/mL 7 days after trauma; unfavorable outcome occurred in 83.3% of these patients and in 11.11% of the other patients (*P* < 0.01).

## 4. Discussion

Revascularization therapy to enhance trophic blood supply is now considered promising for the treatment of patients who suffered TBI, although angiogenesis may be of importance for the secondary brain damages development [1–3]. During this mechanism, pro- and antiangiogenesis factors, which stimulate and maintain angiogenesis, participate and contribute to tissue remodelling after injury. These mediators of angiogenesis are multifunctional and interact with each other, and they may lead to deleterious secondary brain damage or play a neuroprotective role [6]. There are many reports on the expression of proangiogenic factors in the lesions of experimental TBI models and human TBI [7–9], but limited data is available about the antiangiogenic factors following TBI. As one of the most potent endogenous angiogenesis inhibitors, endostatin/collagen XVIII is fundamentally important for regulating vascular permeability and mediating vasorelaxation [15], plays the role as a “stop signal” counteracting the proangiogenic response during wound healing, reduces granulation tissue formation, and causes hemorrhage in wound tissue and severe narrowing of the wound [16]. However, it does not significantly affect the overall wound healing process [17].

Previous clinical and experimental pieces of evidence have proved that endostatin/collagen XVIII was prevailingly expressed and secreted during the early activation of microglia/macrophages after TBI [10–12]. Deininger et al. reported an accumulation of endostatin/collagen XVIII in patients suffering TBI since 36 h up to day 14 and a consequent decrease to day 16 after trauma. In vitro experiments revealed that endostatin expression and release are governed by hypoxia and reactive oxygen intermediates. Zhang et al. observed significant endostatin/microglia accumulation as early as 24 h after TBI which increased steadily up to 96 h during the observation period. Mueller et al. have analyzed the expression of endostatin/collagen XVIII following stab wound injury and detected the maximal endostatin/collagen XVIII(+) monocytic cell numbers at day 14, declining until day 21 after injury. We have analysed the time course of endostatin/collagen XVIII in human CSF following sTBI by ELISA and detected a similar kinetic characteristic compared to the above reports. In our study, a significant increase in the CSF concentrations of endostatin/collagen XVIII was observed as early as 72 h following TBI and extended up to the end of our observation. In addition, our results showed that the cerebrospinal fluid endostatin level closely correlated to the severity of traumatic brain injury. We found that CSF endostatin/collagen XVIII concentration of patients with GCS 3–5 was substantially higher at 72 h after injury and peaked rapidly at day 5 after injury, approximately 1.5 times that in patients with GCS 6–8. It was speculated that the deteriorative changes following increased inflammatory cytokines, reactive oxygen species, and a biphasic response of the vascular system to TBI aggravated the cerebral hypoxia, thereby induced more macrophage and microglia to secrete endostatin to the cerebrospinal fluid. An interesting finding in our study is that CSF level of endostatin/collagen XVIII in extra severe patients has not increased over time, but declined 7 days after trauma. This change is considered to be due to a rapid and extensive apoptosis of reactive macrophages/microglia following the aggravation of the injury.

Endostatin/collagen XVIII has been shown to have a more profound affect on tumor neovasculature. It plays certain inhibiting roles in axon sprouting and neuronal and glial development, although the mechanism of its actions remains undefined. However, the role of endostatin during the tissue remodelling response to CNS injury remains largely elusive. Mueller et al. and Zhang et al. observed that endostatin/collagen XVIII^+^ cells are not only accumulated at the lesion site, in pannecrotic debris zone, and perivascular Virchow-Robin spaces (the drainage route for blood-borne leucocytes), but also at the lesion margin after TBI and spinal cord injury [10, 11, 18]. These areas are prevailingly characterised as regions of developing secondary damage. They speculated that the prolonged endostatin expression might play a role in counteracting the preceding “early” neoangiogenic response after TBI, linked to a “late” CNS-macrophage-mediated secondary injury. However, Deininger et al. considered that endostatin is antagonist in the development of secondary injury following TBI. They believed that long-term surviving brain tissues were in fact characterized by exclusive reactive astrogliosis or glial scarring, frequently lacking the initially formed blood vessels. The increased endostatin expression in areas of vascular pruning and regression following TBI points to a role in the termination of the transient angiogenic response and the maintenance of structure stability of the new vessels. Although the association between endostatin and secondary brain damage is still controversial, we now revealed that this factor's levels in CSF might be useful as predictors of outcome in cases of severe TBI. Using the ROC and AUC, we found a reliable operating point for the 7-day CSF endostatin/collagen XVIII concentration predicting poor prognosis to be 67.29 pg/mL. 

As potential mediator of vascularization, the proangiogenic factor VEGF is upregulated at the lesion site during the period of maximal endothelial proliferation following TBI [8], giving evidence that VEGF also participates in the development of secondary injury. Mechanisms underlying the antiangiogenic ability of endostatin have been proposed to directly abrogate VEGF/VEGF-receptor interaction [19]. Furthermore, VEGF is also induced by H_2_O_2_ and hypoxia and modulated by nitric oxide pathway [20]. Accordingly, it is particularly noteworthy whether there is a link between their expression and secretion during this progress. Compared to VEGF expression in TBI by days 3–6 [4, 5, 8], Deininger et al. observed a time-dependent increase of endostatin/collagen XVIII labeled cells in patients suffering from TBI that peaks at day 15 after injury, approximately a week after VEGF peak expression is observed. It suggests that endostatin plays a role in the modulation of TBI independent of VEGF. Mueller et al. observed the areas of increased numbers of endostatin/collagen XVIII^+^ vessels matched with the districts in which VEGF expression was described by others earlier extending up to approximately 30–100 *μ*m. Shore et al. detected a peak of VEGF levels in CSF of infants and children at 22.4 hours after TBI. But we have not investigated the peak of CSF endostatin/collagen XVIII levels till the end of the observation period. Further investigation is warranted to determine whether this increase is associated with an increase in CSF VEGF and determine the role of VEGF in triggering the increase in CSF endostatin/collagen XVIII concentration.

There are several limitations to our study. First, it is unclear whether CSF endostatin/collagen XVIII concentration from the lumbar subarachnoid space reflects simultaneous brain intracellular or interstitial concentration. Second, the number of patients is small and selectively limits the power to detect potential effects due to differences in endostatin/collagen XVIII concentration, injury severity, and clinical outcome. Third, our analysis is restricted to CSF samples taken from the patients requiring lumbar drainage of CSF. This might have introduced a significant bias in patient selection, and it is therefore difficult to confirm the relationship between the type of TBI and CSF levels of endostatin/collagen XVIII. In addition, the concentration of endostatin/collagen XVIII in the CSF of control group may not represent the normal baseline because of the interference caused by the controls' potential disease of other system. Finally, there were minor variations in the treatment of the TBI patients, although these patients were all treated by the same neurointensive care team using the same standardized protocol.

## 5. Conclusions

We describe the time-dependent increase of endostatin/collagen XVIII levels in CSF of patients with severe TBI that elevated within the first week after injury. This observation supports the hypothesis that endostatin/collagen XVIII plays an important role in the response of the CNS to injury, possibly involving development of secondary brain damage. However, we could not uncover its role in this process. It may provide a theoretical basis for a new pharmacotherapy, selectively attenuating the endostatin/collagen XVIII expression by NO synthase inhibitor, to intervene secondary brain damages. Further studies are also needed to clarify the precise relationship between CSF VEGF and CSF endostatin/collagen XVIII in severe TBI. 

## Figures and Tables

**Table 1:** Clinical characteristics of the controls and patients with severe traumatic brain injury.

Characteristic	Severe TBI (*n* = 30)	Noninjury control (*n* = 20)	*P* value
Age (yrs)*	51.1 ± 6.6	52.1 ± 8.8	0.6483
Gender (male/female)^†^	20/10	13/7	1.0
GCS on admission	6.3 ± 1.5	NA	NA
Mechanism of injury		NA	NA
MVA	22 (73.3)		
Fall	6 (20)		
Heavy strikes/assault	2 (6.7)		
Types of lesions		NA	NA
Contusions/lacerations	10 (33.3)		
ICHs	15 (50)		
BSI/DAI	5 (16.7)		

Data presented as mean ± standard deviation or *n* (%).

TBI: traumatic brain injury; NA: not applicable; MVA: motor vehicle accident; GCS: Glasgow Coma Score; ICHs: Intracranial hematomas; BSI: brain stem injury; DAI: diffuse axonal injury.

*t-test.

^†^Fisher's exact test.

**Table 2:** Comparison of CSF endostatin levels between survival and death group (pg/mL).

	24 h after injury	72 h after injury	5 d after injury	7 d after injury
Survival (*n* = 24)	31.30 ± 2.91	43.82 ± 4.69	50.82 ± 6.59	60.94 ± 7.78
Death (*n* = 6)	37.20 ± 8.14	69.52 ± 11.24	108.32 ± 14.95	78.83 ± 16.33
*P* value	0.412	0.026	0.001	0.317

Values are expressed as mean ± standard error of the mean.

*P* value obtained by Mann-Whitney test for the difference between the two groups.

**Table 3:** CSF concentrations of endostatin in 30 severe TBI patients per 6-month outcome (pg/mL).

	24 h after injury	72 h after injury	5 d after injury	7 d after injury
Favorable* (*n* = 18)	30.13 ± 3.73	44.29 ± 5.55	49.36 ± 7.47	46.83 ± 5.51
Unfavorable^†^ (*n* = 12)	36.01 ± 4.28	55.98 ± 8.19	81.75 ± 12.89	91.06 ± 12.16
*P* value	0.316	0.230	0.043	0.005

Values are expressed as mean ± standard error of the mean.

*Patients with a GOS score of 4 or 5.

^†^Patients with a GOS score of 1, 2, or 3.

*P* value obtained by Mann-Whitney test for the difference between the two groups.

**Table 4:** Area under the ROC curve of the prognosis at 6 months after trauma.

CSF endostatin/collagen XVIII	AUC	Standard error	*P* value	95% CI
24 h after injury	0.606	0.104	0.330	0.403–0.810
72 h after injury	0.653	0.107	0.162	0.444–0.862
5 d after injury	0.704	0.100	0.063	0.508–0.900
7 d after injury	0.843	0.072	0.002**	0.701–0.984

AUC: area under the ROC curve; CI: confidence interval.

**P < 0.01 marked statistical significance.
